# A Novel Copper(II) Indenoisoquinoline Complex Inhibits Topoisomerase I, Induces G2 Phase Arrest, and Autophagy in Three Adenocarcinomas

**DOI:** 10.3389/fonc.2022.837373

**Published:** 2022-02-24

**Authors:** Caroline Molinaro, Nathalie Wambang, Till Bousquet, Anne-Sophie Vercoutter-Edouart, Lydie Pélinski, Katia Cailliau, Alain Martoriati

**Affiliations:** ^1^ Univ. Lille, CNRS, UMR 8576-UGSF-Unité de Glycobiologie Structurale et Fonctionnelle, Lille, France; ^2^ AGAT Laboratories, Intertek, Montréal, QC, Canada; ^3^ Univ. Lille, CNRS, Centrale Lille, Univ. Artois, UMR 8181-UCCS-Unité de Catalyse et Chimie du Solide, Lille, France

**Keywords:** indenoisoquinoline, copper(II) complex, adenocarcinoma, topoisomerase, cell cycle, autophagy

## Abstract

Topoisomerases, targets of inhibitors used in chemotherapy, induce DNA breaks accumulation leading to cancer cell death. A newly synthesized copper(II) indenoisoquinoline complex WN197 exhibits a cytotoxic effect below 0.5 µM, on MDA-MB-231, HeLa, and HT-29 cells. At low doses, WN197 inhibits topoisomerase I. At higher doses, it inhibits topoisomerase IIα and IIβ, and displays DNA intercalation properties. DNA damage is detected by the presence of γH2AX. The activation of the DNA Damage Response (DDR) occurs through the phosphorylation of ATM/ATR, Chk1/2 kinases, and the increase of p21, a p53 target. WN197 induces a G2 phase arrest characterized by the unphosphorylated form of histone H3, the accumulation of phosphorylated Cdk1, and an association of Cdc25C with 14.3.3. Cancer cells die by autophagy with Beclin-1 accumulation, LC3-II formation, p62 degradation, and RAPTOR phosphorylation in the mTOR complex. Finally, WN197 by inhibiting topoisomerase I at low concentration with high efficiency is a promising agent for the development of future DNA damaging chemotherapies.

## Introduction

Adenocarcinomas are the most diagnosed cancers. Among them, breast and cervix, respectively the first and fourth most represented cancers in women, and colorectal cancers the second and third most represented cancers respectively in women and men (1). Current treatments include chemotherapy with agents that generate DNA damage to trigger cancer cell division arrest and associated programmed cell death of tumours (2, 3).

Topoisomerases (Top) regulate DNA topology during replication, transcription, and chromosomal segregation (4–6). To relieve torsional strain, these DNA-interacting enzymes cleave one or two DNA strands before the religation step (7, 8). Human Top are subdivided into three subgroups including IA (Top3α and Top3β), IB (Top1 nuclear and Top1 mitochondrial), and IIA (Top2α and Top2β), type I Top cause single-strand breaks (SSB) while type II Top generate double-strand breaks (DSB) (9). In anticancer therapy, inhibition of Top allows DNA cleavage, prevents the religation reaction, and leaves cancer cells with DNA breaks. Top1 and Top2 are mainly targeted due to their overexpression in many cancers including breast, cervix, and colorectal cancers (10–13). The increased quantity and activity of Top in highly dividing cells directly correlate with positive responses to Top inhibitory treatments (12, 14, 15). The primary cytotoxic lesions in cancer cells result from collisions between the trapped Top and the replication forks (16–18). DNA breaks further trigger the activation of DNA Damage Response (DDR) pathways, leading to cell cycle arrest and to death if DNA damage is too severe (19, 20). The DDR pathways start with the recruitment and the phosphorylation of histone H2AX on serine 139 (γH2AX) by phosphoinositide 3-kinase related kinase family members ATM, ATR, and DNA-PK (21, 22). Consecutively, Chk1 and Chk2 kinases are activated, inhibit phosphatase Cdc25 (23), and induce a cell cycle arrest followed in most cases by apoptosis (20).

Top inhibitors display different action mechanisms. Poisons target the DNA/topoisomerase cleavage complex, form a ternary complex (interfacial inhibition) inhibiting DNA religation, and result in persistent DNA breaks (24). Catalytic inhibitors either intercalate into DNA in the Top fixation site or are ATP competitors or hydrolysis inhibitors to provoke an antineoplastic effect (25). A small number of Top inhibitors are approved for clinical use. The Top2 poison doxorubicin and its isomer epirubicin from the anthracycline family are first-line antineoplastic agents used against many different types of solid tumors, leukemias, and lymphomas (26, 27), with main side effects including cardiotoxicity and t-AML (treatment-related acute myelogenous leukemia) (28–30). At high doses (up to 10 µM), doxorubicin becomes a DNA intercalator and contributes to increase DNA breaks (31, 32). Top2 poison etoposide (VP-16) also induces t-AML (9). The Top1 poison camptothecin derivatives, topotecan and irinotecan, are used to treat solid tumors including ovary, cervix, pancreatic, lung, and colorectal cancers (33). However, their use in chemotherapy is limited by their instability, the need for long-term chemotherapies, and by severe side effects including hematotoxicity, vomiting and diarrhea (34). Unlike camptothecins, the Top1 inhibitors indenoisoquinolines are chemically stable, are not substrates for drug efflux transporters and as such are promising Top inhibitors (35, 36). Indenoisoquinoline derivatives (LMP400, LMP776, and LMP744) are in phase I/II clinical trials (35, 36).

Since the discovery of platinum anticancer properties and the use of cisplatin, a platinum-based alkylating agent, and its derivatives in chemotherapy (37–39), other metal-based drugs have been designed and developed for their cytotoxic effects on tumour cells (40–42). Transition metals from the d-block of the periodic table (groups 3 to 12) (43–46) are particularly suitable for this purpose as they adopt a wide variety of coordination geometries (47). Among them, copper modifies the backbone of the complexed ligand and grants better DNA affinity (48–50). Copper derivatives interact with DNA using noncovalent interactions with the major or the minor DNA grooves, intercalation, or electrostatic binding to enhance DNA damage, and display antitumor activity (51). Some copper complexes inhibit either or both Top1 and Top2 and results in severe DNA damage, cell cycle arrest, and death in cancer cells (52, 53).

As a part of an ongoing effort to develop new efficient anticancer organometallic drugs and to palliate limitations in drug resistances and/or side effects, the synthesis of a novel copper(II) complex of indenoisoquinoline ligand, named WN197, is established based on previous studies (54, 55). This organo-copper complex effects were investigated on breast triple-negative MDA-MB-231, cervix HeLa, and colon HT29 cell lines representative of three most prevalent adenocarcinomas, and associated with poor prognostics. WN197 exerts a specific cytotoxic effect at low concentration (IC_50_ below 0.5 µM) on the three cell lines and significantly below the value of human non-tumorigenic epithelial cell line MCF-10A (IC_50_ 1.08 µM). WN197 acts as a Top1 poison and displays DNA intercalation properties. The action mechanism of WN197 is further deciphered to bring insights into its efficiency. DNA damage is detected by the presence of a rapid increase in nuclear phosphorylated H2AX (after 30 min of treatment with 0.5 µM) and the main DDR kinases are activated by phosphorylations. Cell cycle arrest in the G2 phase is confirmed by the inhibitory phosphorylation of Cdk1 on tyrosine 15, an accumulation of cyclin B, and the unphosphorylated form of histone H3. Furthermore, the cell cycle is halted in G2 by inhibitory phosphorylation of Cdc25C on serine 216 associated with a binding to the 14.3.3 chaperon. Cancer cells halt in G2, die by autophagy detected through an increase in Beclin-1, and a decrease in the LC3-I/LC3-II ratio and the p62 marker. Moreover, the RAPTOR component in the mTORC1 complex is phosphorylated on serine 792, a feature of autophagic-induced cell death.

## Materials and Methods

### Chemical Reagents and Materials

All commercial reagents and solvents were used without further purification. Cisplatin is purchased from Alfa Aesar (Heysham, UK); rapamycin from Abcam (Cambridge, UK); doxorubicin, nocodazole and DMSO from Sigma-Aldrich (Saint-Quentin-Fallavier, France). Stock solutions were prepared in DMSO. Melting points were determined with a Barnstead Electrothermal (BI 9300) capillary melting point apparatus and are uncorrected. Elemental analyses were performed with a varioMICRO analyser. Thin layer chromatography (TLC) was carried out on aluminium-baked (Macherey-Nagel GmbH, Düren, Germany) silica gel 60. Column chromatography was performed on silica gel (230-400 mesh). The electronic absorption spectra were acquired on a UV-Vis double beam spectrophotometer SPECORD^®^ PLUS (Analytik Jena GmbH, Germany). The molar conductance measurement was carried out using a CDRV 62 Tacussel electronic bridge, employing a calibrated 10^-2^ M KCl solution and 10^-3^ M solutions of compounds in DMSO. Purities of all tested compounds were ≥95%, as estimated by HPLC analysis. High Resolution Mass Spectrum (HR-MS) was measured at REALCAT (Université de Lille) on a Synapt G2Si (Waters) equipped with an ion mobility cell.

### WN197 Copper(II) Indenoisoquinoline Complex Synthesis

WN170 was synthesized according to the literature procedure (56). To a solution of WN170 (160 mg, 0.443 mmol) in dry methanol (8 mL) was added dropwise a solution of CuCl_2_ (59 mg, 0.443 mmol) in MeOH (7 mL). After stirring at room temperature for 10 h, the reaction mixture was filtered off to yield an orange precipitate which was washed with MeOH and dried under vacuum (8 h at 100°C). Yield: 132 mg (70%). Decomposition at 194°C. Anal. Calcd. for C_44_H_54_Cl_2_CuN_6_O_8_ (%): C, 56.86; H, 5.86; N, 9.04: Found C, 56.76; H, 5.89; N, 9.22. FT-IR (neat) (νmax, cm^-1^): 1650 (C=O), 1549 (C=C), 490 (Cu-N). UV-vis in DMSO-H_2_O (19/01), λ/nm (ϵ/M^−1^cm^−1^): 625 (156), 463 (4500) (9800), 353 (17620), 350 (18100), 328 (16440). Λ_M_ (1 mM, DMSO) (S cm^2^ mol^-1^): 24. HRMS (ESI) m/z: calcd for [M]^+^ C_44_H_46_ClCuN_6_O_4_ 820.2565; Found 820.2332.The equations should be inserted in editable format from the equation editor.

### Cell Culture

HeLa, MDA-MB-231, HT-29 and MCF-10A cell lines originate from ATCC (Manassas, VA, USA), and were maintained at 37°C in a humidified atmosphere with 5% CO_2_ in DMEM medium (Lonza, Basel, Switzerland) supplemented with 10% fetal bovine serum (Dutscher, Dernolsheim, France), 1% Zell Shield (Dutscher, Bernolsheim, France) and 1% non-essentials amino-acids (Lonza, Basel, Switzerland). MCF-10A were maintained in MEBM medium (Lonza, Basel, Switzerland) supplemented with MEGM (Lonza, Basel, Switzerland). All cell lines culture media were added with 1% Zell Shield (Dutscher, Bernolsheim, France).

### Cell Viability Assay

Cell viability was determined using CellTiter 96^®^ AQ_ueous_ One Solution Cell Proliferation Assay (MTS, Promega, Charbonnières-les-Bains, France). 2.10^3^ cells well were seeded in 96-well plate for 24 h before treatment with 0 to 100 µM of WN197, WN170 or cisplatin for 72 h. After a 2 h incubation with 20 µL of CellTiter solution at 37°C in 5% CO_2_, the production of reduced MTS (3-(4,5-dimethylthiazol-2-yl)-5-(3-carboxymethoxyphenyl)-2-(4-sulfophenyl)-2H-tetrazolium) in formazan was measured at 490 nm (SPECTROstar Nano, BMG LABTECH, Ortenberg, Germany). IC_50_ were calculated using GraphPad Prism V6.0 software. Statistical differences between WN197 and WN170 were ascertained by a Student *t*-test (**p<0.01 and ****p<0.0001).

### Immunofluorescence for Nuclei *Foci*

2.10^5^ cells seeded on glass coverslips were treated with 0.5 µM of WN197 or WN170, 5 µM of doxorubicin, 20 µM of cisplatin as positive controls, or 0.1% DMSO as a solvent control for 30 min or 24 h. Fixation was performed with 4% paraformaldehyde (Sigma-Aldrich, Saint-Quentin-Fallavier, France) for 5 min and followed by permeabilization with 0.1% Triton in PBS (Sigma-Aldrich, Saint-Quentin-Fallavier, France) for 10 min and saturation of unspecific sites with 1% BSA in PBS (Sigma) for 1 h at room temperature. Anti-γH2AX mouse antibody (S139, 1:1000, Cell Signalling, by Ozyme, Saint-Cyr-L’École, France) was incubated overnight at 4°C, washed 3 times with 1% BSA/PBS. Cells were incubated with secondary anti-mouse IgG (Alexa Fluor^®^ 488, 1:2000, Thermo-Fisher Scientific Biosciences GMBH, Villebon-sur-Yvette, France) for 1 h at room temperature in the dark, washed 3 times before nuclei were stained with DAPI (6-diamidino-2-phenylindole, 1 µg/mL, Molecular Probes, by Thermo Fisher Scientific Biosciences GMBH, Villebon-sur-Yvette, France). Images were captured under a Leica fluorescent microscope, and γH2AX *foci* were counted with ImageJ (Fiji Software, v1.52i) on 30 cells from 3 independent experiments and quantified with GraphPad Prism V6.0 software. Statistical significances (mean ± SD) were performed by a two-way ANOVA followed by Dunnett’s multiple comparison test (**p<0,01; ***p<0,001; ****p<0,0001).

### Electrophoresis and Western Blot

7.5.10^5^ cells were seeded for 24 h and treated with 0.5 µM of WN197 or WN170, 20 µM of cisplatin, 5 µM of doxorubicin, or 0.1% DMSO (solvent control). After 24 h, they were lysed in RIPA buffer (1% Triton X-100; 50 mM TRIS-HCl pH 4; NP40 2%; 0.4% Na-deoxycholate; 0.6% SDS; 150 mM NaCl; 150 mM EDTA; 50 mM NaF) supplemented with 1% of protease inhibitor cocktail (Sigma-Aldrich, Saint-Quentin-Fallavier, France) and phosphatase inhibitors (Roche SAS by Merck, Kenilworth, NJ, USA).

For cytochrome C analysis, 7.5.10^5^ cells were seeded for 24 h and treated for 3 h, 16 h, 24 h or 48 h with 0.5 µM of WN197, and for 24 h or 48 h with 5 µM of doxorubicin as positive control. Cells were lysed in a glass grinder at 4°C in homogenization buffer (25 mM MOPS at pH 7.2, 60 mM β−glycerophosphate, 15 mM para-nitrophenylphosphate, 15 mM EDTA, 15 mM MgCl2, 2 mM DTT, 1 mM sodium orthovanadate, 1 mM NaF, 1 mM phenylphosphate, 10 μg/mL leupeptin, 10 μg/mL aprotinin, 10 μg/mL soybean trypsin inhibitor, 10 μM benzamidine).

Samples were centrifuged for 10 min at 12,000 G and protein concentration of supernatants were determined using the Bradford assay (BioRad, Marnes-la-Coquette, France) at 595 nm (SPECTROstar Nano, BMG LABTECH, Ortenberg, Germany). Proteins were denatured in 2X Laemmli buffer (65.8 mM TRIS-HCl pH 6.8; 26.3% glycerol; 2.1% SDS; 0.01% bromophenol blue; 4% β-mercaptoethanol, BioRad, Marnes-la-Coquette, France) at 75°C for 10 min. 15 µg of proteins were separated on 4-20% SDS PAGE gels (mini protean TGX, BioRad, Marnes-la-Coquette, France), for 1 h at 200 V in denaturing buffer (0.1% SDS; 0.3% TRIS base; 1.44% glycine). Proteins were transferred onto nitrocellulose membrane (Amersham Hybond, Dutscher, Bernolsheim, France) by wet transfer (0.32% TRIS; 1.8% glycine; 20% methanol, Sigma-Aldrich, Saint-Quentin-Fallavier, France), for 1 h at 100 V. Membranes were saturated with 5% low fat dry milk in TBS added with 0.05% Tween (Sigma-Aldrich, Saint-Quentin-Fallavier, France), and incubated overnight at 4°C with specific primary antibodies: rabbit polyclonal antibodies were against ATM (Cell Signaling technology (CST, by Ozyme, Saint-Cyr-L’École, France), 1/1000), ATR (CST, 1/750), phosphorylated ATR (S428, CST, 1/1000), Beclin-1 (CST, 1/800), Cdc25C (CST, 1/1500), phosphorylated Cdc25C (S216, CST, 1/1000), phosphorylated Cdk1 (Y15, CST, 1/1500), phosphorylated Chk1 (S317, CST, 1/1000), phosphorylated Chk2 (T68, CST, 1/1000), cleaved caspase 3 (CST, 1/1000), phosphorylated H2AX (S139, CST, 1/750), histone H3 (CST, 1/1000), phosphorylated H3 (S10, CST, 1/1000), phosphorylated p53 (S15, CST, 1/1000), p53 (CST, 1/1000), p21 (CST, 1/1000), LC3 (CST, 1/50), mTOR (CST, 1/1200), RAPTOR (CST, 1:1500), phosphorylated RAPTOR (S792, CST, 1/1000); mouse monoclonal antibodies against phosphorylated ATM (S1981, Santa Cruz Biotechnology (SCB), Santa Cruz, CA, USA, 1/200), Chk1 (SCB, 1/1000), Chk2 (SCB, 1/200), Cdk1 (CST, 1/1000), 14-3-3 (SCB, 1/1000), cyclin B2 (CST, 1/1500), p62 (SCB, 1/100); goat polyclonal antibodies against β-actin (SCB, 1/1200); and cocktail antibodies against cleaved PARP (Abcam, Cambridge, UK, cell cycle and apoptosis cocktail, 1/1500). After three washes of 10 min in TBS-Tween, nitrocellulose membranes were incubated 1 h with the appropriate horseradish peroxidase-labeled secondary antibodies: anti-rabbit or anti-mouse antibodies (Invitrogen, by Thermo Fisher Scientific Biosciences GMBH, Villebon-sur-Yvette, France, 1/30,000) or anti-goat antibodies (SCB, 1/30,000). Secondary antibodies were washed in TBS-Tween three times for 10 min and the signals were revealed with a chemiluminescent assay (ECL Select, GE Healthcare, Dutscher, Bernolsheim, France) on hyperfilms (Amersham hyperfilm MP, Dutscher, Bernolsheim, France). β-actin or histone H3 were used as loading controls. Signals were quantified with Image J (Fiji Software, v1.52i), and normalized to respective loading control. The means of 3 independent experiments were calculated.

### *In Vitro* Activities of Human Topoisomerases I and II

Topoisomerase activities were examined in assays based on the relaxation of a supercoiled DNA into its relaxed form. Topoisomerase I (Top1) activity was performed using the drug screening kits protocol (TopoGEN, Inc., Buena Vista, CO, USA). The reaction mixture was composed of supercoiled pHOT1 DNA (250 ng), 10X TGS buffer (10 mM Tris-HCl pH 7.9, 1 mM EDTA), 5 units of Top1, a variable amount of compound to be tested, and a final volume adjusted to 20 µL with H2O. WN197 was tested at concentrations ranging from 0.2 to 2 µM. Camptothecin (10 µM) was used as a positive control (poison inhibitor of Top1 activity), etoposide (100 µM) as negative control (inhibitor of Top2 activity), and 1% DMSO alone as vehicle control. Relaxed pHOT1 DNA (100 ng) was used as migration control. The addition of proteinase K (50 µg/mL) for 15 min at 37°C allowed Top1 degradation to visualize the cleavage products (nicked DNA). Reaction products were separated by electrophoresis in a 1% agarose gel containing ethidium bromide (0.5 µg/mL) for 1 h at 100 V in TAE (Tris-Acetate-EDTA; pH 8.3) buffer.

Topoisomerase II Relaxation Assay Kit (Inspiralis, Inc., Norwich, UK) was used to measure topoisomerase II (Top2) activity. The reaction mixture was composed of supercoiled pBR322 DNA (1 µg), 10X assay buffer (50 mM Tris-HCl (pH 7.5), 125 mM NaCl, 10 mM MgCl2, 5 mM DTT, 100 µg/mL albumin), 30 mM ATP, 5 units of Top2α or Top2β, variable amount of compound to be tested, and a final volume adjusted with H2O to 30 µL. Etoposide (VP-16, 100 µM) was used as positive control, and camptothecin (10 µM) as negative control. The mixtures were incubated at 37 °C for 30 min and the reactions stopped by the addition of 5 µL 10% SDS. Reaction products were separated by electrophoresis in a 1% agarose gel for 1 h at 100 V in TAE buffer, and stained with ethidium bromide (0.5 µg/mL) for 15 min. After destaining in water, the DNA migratory profiles were visualized under UV light (ChemiDocTM XRS+, BioRad, Marnes-la-Coquette, France).

### Melting Temperature Measurement

Melting temperatures were obtained as described (54, 55). 20 µM solutions of WN170 or WN197 were prepared in 1 mL of BPE buffer (2 mM NaH_2_PO_4_, 6 mM Na_2_PO_4_, 1 mM EDTA, pH 7.1) in the presence or not of 20 µM DNA from calf thymus (42% GC bp, Merck, Kenilworth, NJ, USA). Absorbances were measured at 260 nm (Uvikon 943 coupled to Neslab RTE111) every minute over the range of 20 to 100°C with an increment of 1°C per minute. All spectra were recorded from 230 to 500 nm. Tested compound results are referenced against the same DNA concentration in the same buffer. The Tm values were obtained from the first derived plots.

### Ethidium Bromide Competition Test

Fluorescence titrations were determined as described (54, 55). Ethidium bromide/WN170 or WN197 molar ratio of 12.6/10 at concentrations ranging from 0.05 to 10 µM were used in a BPE buffer (pH 7.1). The excitation wavelength was set at 546 nm and the emission was monitored over the range of 560 to 700 nm (SPEX Fluorolog). IC_50_ values for ethidium bromide (EB) displacement were calculated using a fitting function incorporated into GraphPad Prism 6.0 software. The apparent binding constants were calculated using the equation K_app_ = (1.26 (K_app_(EB)/IC_50_) with K_app_(EB) =10^7^ M^−1^ and IC_50_ in μM.

### Flow Cytometry

7.5.10^5^ cells plated for 24 h were treated with 0.5 µM WN197 or WN170, 20 µM of cisplatin (S phase arrest control), 83 nM of nocodazole (M phase arrest control), or 0.1% DMSO (solvent control). For the dose titration experiments, cells were treated for 24 h with increasing concentrations of WN197. For kinetic experiments, cells were treated with 0.5 µM of WN197 or WN170 from 4 to 48 h. Cells were detached using trypsin (Biowest, Nuaillé, France), centrifuged at 1,000 G for 10 min, resuspended in PBS, and fixed with 70% ethanol at -20°C for 24 h, before they were centrifugated (1,000 G, 10 min), resuspended in PBS, and treated for 15 min at room temperature with RNase (200 µg/mL, Sigma). Finally, incubation with propidium iodide (10 µL/mL, Molecular Probes, by Thermo Fisher Scientific Biosciences GMBH, Villebon-sur-Yvette, France) at 4°C for 30 min was performed before flow cytometry (BD FACSCalibur, Becton Dickinson, Le Pont-de-Claix, France) analysis. For each sample, 10,000 events (without cell doublets and cellular debris) were considered. The cell cycle repartition was analyzed with Graphpad Prism V6.0 software. Statistical significances (mean ± SD) were determined by two-way ANOVA followed by Dunnett’s multiple comparison test (****p<0,0001).

### Immunoprecipitation

Cell lysates were obtained as described in the Western blot section. Samples were pre-cleared with protein A sepharose (20 μL of 50% beads/200 μL of cell lysate, Sigma-Aldrich, Saint-Quentin-Fallavier, France) for 1 h at 4°C under gentle rocking. After brief centrifugation, supernatants were incubated with antibodies against 14.3.3 (Santa Cruz Biotechnology, Santa Cruz, CA, USA, 1/200), Cdc25C (Thermo Fisher Scientific Biosciences GMBH, Villebon-sur-Yvette, France, 1/200) or mTOR (CST, 1/200) at 4°C for 1 h under rotation and followed by incubation with protein A sepharose (20 μL of 50% bead slurry, Sigma-Aldrich, Saint-Quentin-Fallavier, France) for 1 h at 4°C under rotation. Samples were rinsed 3 times with RIPA buffer. Pellets were collected by brief centrifugation, resuspended in 2X Laemmli buffer, and heated at 100°C for 10 min before SDS-PAGE and Western blots were performed.

## Results

### Organocopper Synthesis

**Figure 1:**
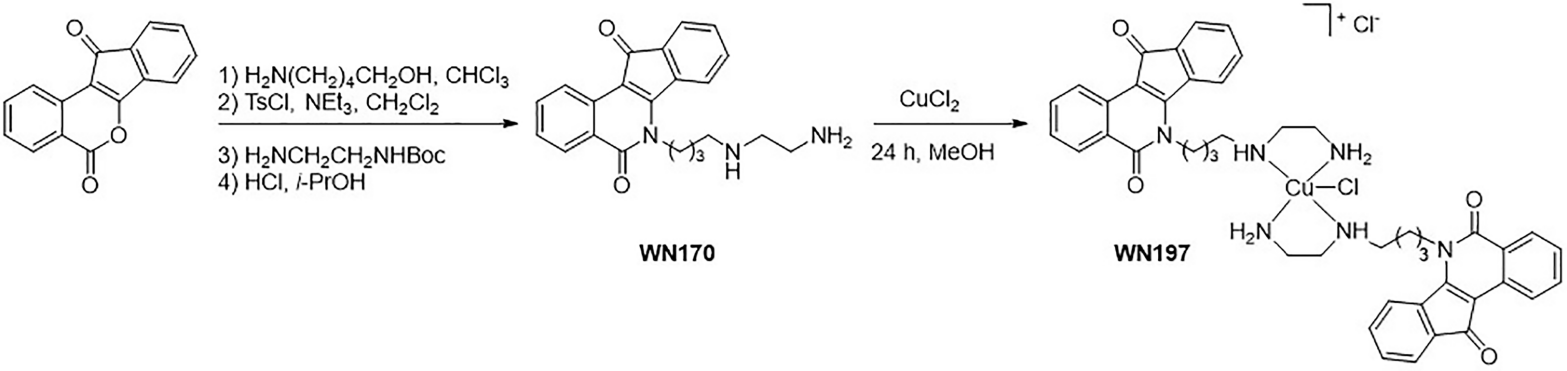
WN197 synthesis.

The synthesis of WN197 is described in **Figure 1**. Indenoisoquinoline WN170 was first obtained in a four-step reaction. Condensation of the benzo[d]indeno[1,2-b]pyran-5,11-dione with a primary aminoalcohol was followed by tosylation of the alcohol function. The substitution of the tosyl group by the protected ethylenediamine and the consecutive deprotection of the Boc group led to WN170 in 68% global yield. Complex WN197 was then synthesized by reacting methanolic solutions of indenoisoquinoline derivative WN170 and CuCl_2_. After purification, WN197 was obtained in 70% yield.

### WN197 Displays a Cytotoxic Activity on Three Adenocarcinoma Cell Lines at Low Doses

Cells viability was assayed on the triple-negative breast cancer cells (MDA-MB-231), the cervix cancer cells (HeLa), and the colorectal cancer cells (HT-29) (**Table 1**). IC_50_ obtained are respectively 0.144 µM, 0.22 µM, and 0.358 µM for WN197 below the cisplatin IC_50_ values ranging from 10 to 40 µM. The copper-free indenoisoquinoline ligand, WN170, affected cell viability at higher doses (0.875 µM for MDA-MB-231, 0.630 µM for HeLa, and 0.479 µM for HT-29 cells), showing that the presence of the copper metal significantly enhances the anticancer effect of the indenoisoquinoline core for MDA-MB-231 and HeLa cell lines. A significantly higher IC_50_ (1.080 µM) is obtained on MCF-10A compared to the adenocarcinoma cell lines (**Table 2**).

**Table 1:** Half maximal inhibitory concentrations (IC_50_ in μM) for cell survival.

	MDA-MB-231	HeLa	HT-29
WN197	0.144 ± 0.01	0.220 ± 0.01	0.358 ± 0.07
WN170	0.875 ± 0,01	0.630 ± 0.09	0.479 ± 0.07
Cisplatin	33.802 ± 1.27	19.287 ± 5.323	21.313 ± 7.475
*Statistical difference (WN197/WN170)*	****	**	*ns*

Data are expressed as the mean ± SD of three independent experiments. Statistics were based on Student’s t-test of the difference between WN197 and WN170; ns, non-significative, **p<0.01 and ****p<0.0001.

**Table 2:** Half maximal inhibitory concentrations (IC_50_ in μM) for cell survival of MCF-10A.

Compound	IC_50_ (µM)
WN197	1.080 ± 0.037
Cisplatin	14.218 ± 7.157
*Statistical difference (WN197 on adenocarcinomas vs. on MCF-10A)*	***

Data are expressed as the mean ± SD of three independent experiments. Statistics were based on Student’s t-test of the difference between WN197 IC_50_ on adenocarcinomas and MCF-10A; ***p<0.001.

### WN197 Induces DNA Damage

**Figure 2:**
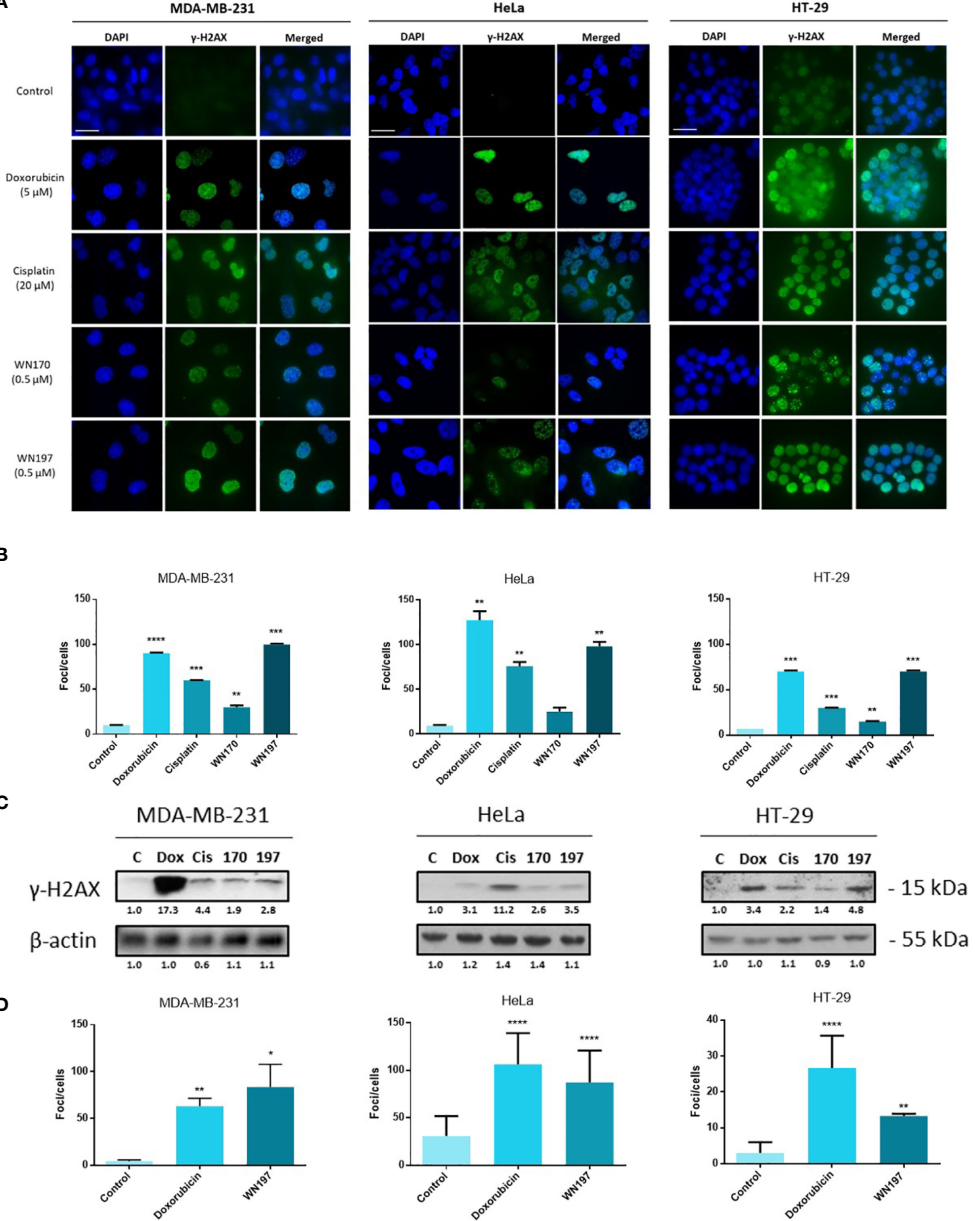
The copper complex WN197 induced DNA damage in cancer cells. MDA-MB-231, HeLa and HT-29 cells were treated with DMSO (0.5%, solvent control), doxorubicin (5 µM, Top2 inhibitor inducing DNA breaks), cisplatin (20 µM, alkylating agent inducing DNA breaks), WN170 (0.5 µM, indenoisoquinoline without metal) or WN197 (0.5 µM). **(A)** Immunofluorescence of the DNA breaks marker γH2AX was visualized as green *foci* in nuclei stained with DAPI (blue) on a Leica fluorescent microscope 24 h after treatments. Images were representative of three independent experiments. Scale bar: 20 µm **(B)** Quantification of γH2AX *foci* number per cells. **(C)** Western blot analysis of γH2AX 24 h after treatments. β-actin was used as a loading control and relative γH2AX level was quantified by densitometry using Image J (Fiji Software, v1.52i). **(D)** Quantification of γH2AX *foci* number per cells 30 min after treatments, based on immunofluorescence experiments. In B and D, data were expressed as the mean ± SD for 30 nuclei of three independent experiments. Statistical analyses were based on a two-way ANOVA followed by a Dunnett’s test (*p<0.05, **p<0,01, ***p<0,005 and ****p<0,001).

To determine whether WN197 affects DNA integrity, detection of γH2AX DNA break marker was performed by immunofluorescence. γH2AX *foci* were visualized in the nucleus at 0.5 µM of WN197, a concentration close to the IC_50_ determined previously, in MDA-MB-231, HeLa, and HT-29. After 24 h of treatment, the average number of γH2AX *foci* per cell were respectively 99, 98, and 70 for MDA-MB-231, HeLa, and HT-29 cells (**Figure 2A**). The number of γH2AX *foci* was close to the result obtained for the Top2 inhibitor, doxorubicin, (average of 95 *foci* per cell), and higher than the number of γH2AX *foci* triggered by an alkylating agent, cisplatin (average of 55 *foci* per cell). WN197 induced more DNA damage than the indenoisoquinoline WN170 (average of 23 *foci* per cell). Controls with DMSO solvent showed a low number of *foci* (average of 9 *foci* per cell for the 3 adenocarcinomas) compared to treated conditions (**Figure 2B**).

These results were further confirmed by Western blot analysis (**Figure 2C**). Untreated cells showed a low γH2AX signal while a strong signal was observed after doxorubicin, cisplatin, and WN197 treatments. As observed by immunofluorescence, the γH2AX signal is weaker in the WN170 condition compared to the WN197 condition, indicating that the WN197 compound induces more DNA damage than WN170 at the same concentration (0.5 µM).

*Foci* were detected as soon as 30 min after treatment (**Figure 2D**). The number of γH2AX *foci* was close to the result obtained at 24 h with an average of *foci* per cell of 84, and 87 for MDA-MB-231, HeLa, and lower to 13 for HT-29 cells after WN197 treatment.

### WN197 Is a Concentration-Dependent Topoisomerase Inhibitor

**Figure 3:**
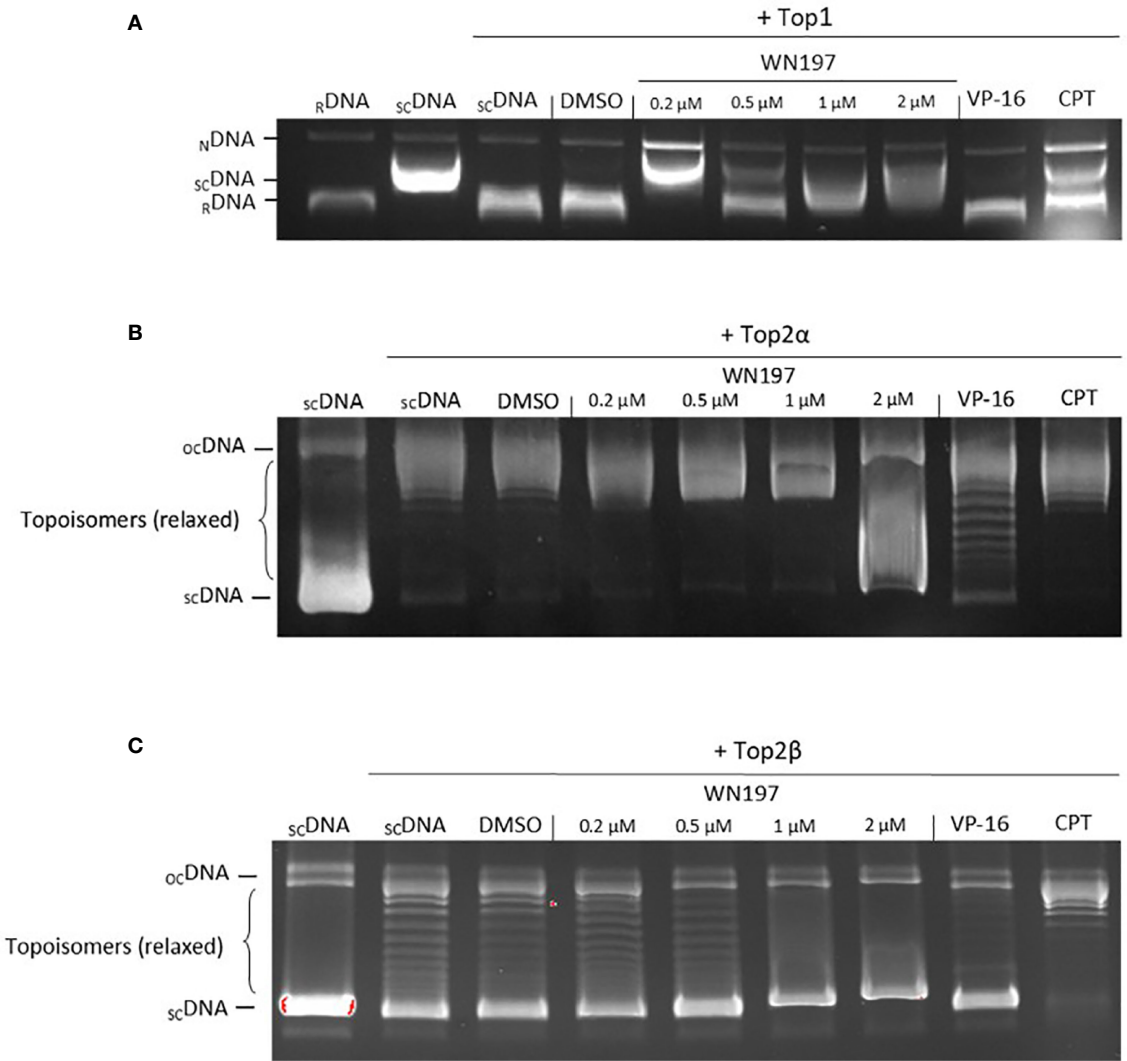
WN197 inhibited human topoisomerase activity in a dose-dependent manner. **(A)** Top1 activity was determined by *in vitro* assays after addition of either DMSO (5%, solvent control, lane 4), WN197 at different concentrations (0.2, 0.5, 1 and 2 µM, lanes 5-8), etoposide (VP-16, 50 µM; Top2 poison, lane 9) the negative control of Top1 activity inhibition, or camptothecin (CPT, 10 µM; Top1 poison, lane 10) the positive control of Top1 activity inhibition. Relaxed DNA (_R_DNA, lane 1) or supercoiled DNA (_SC_DNA, lane 2) were used as migration controls. _SC_DNA was used in all other reactions in presence of Top1. The Top1 activity control allowing the relaxation of _SC_DNA is in lane 3. The addition of proteinase K allowed detection of nicked DNA (_N_DNA), a witness of the single-strand broken DNA stabilization by a topoisomerase poison. **(B)** Top2α activity inhibition assay. Migration control of supercoiled DNA (_SC_DNA) was performed in lane 1. Top2α was present in all other reactions. The Top2α activity control for the relaxation of _SC_DNA is in lane 2, the first band corresponds to the transitional open circular DNA (_OC_DNA) and topoisomers correspond to the relaxed DNA. DMSO (5%, solvent control) in lane 3, WN197 (concentrations of 0.2, 0.5, 1 and 2 µM) in lanes 4-7, etoposide (VP-16, 50 µM; Top2 poison) in lane 8, and camptothecin (CPT, 10 µM; Top1 poison) in lane 9. **(C)** Top2β activity inhibition assay. Migration control of _SC_DNA was performed in lane 1. Top2β was present in all other reactions. The Top2β activity control for the relaxation of _SC_DNA is in lane 2, DMSO (5%, solvent control) in lane 3, WN197 (concentrations of 0.2, 0.5, 1 and 2 µM) in lanes 4-7, etoposide (VP-16, 50 µM; Top2 poison) in lane 8, and camptothecin (CPT, 10 µM; Top1 poison) in lane 9. In **(A–C)** after topoisomerase reactions, DNA was run in a 1% agarose gel, stained with ethidium bromide (0.5 µg/mL), and visualized under UV light.

To determine whether the Cu(II)-complex WN197 is a topoisomerase inhibitor, *in vitro* human topoisomerase activity tests were realized. The topoisomerase I (Top1) test relies on the ability of Top1 to relax supercoiled DNA, and the absence of relaxed DNA implies inhibition of Top1 activity. In the presence of Top1, supercoiled DNA showed a relaxed profile (**Figure 3A**). Camptothecin, a well-known Top1 inhibitor, disturbed DNA relaxation in the reaction, and part of the DNA remained supercoiled. Increasing doses of WN197 from 0.2 to 2 µM showed a decrease quantity of relaxed DNA, indicating disruption of Top1 activity. The solvent control, DMSO, and VP-16 (etoposide, a Top2 inhibitor) displayed no effect on Top1-induced DNA relaxation showing no inhibitory effect on Top1 activity.

Top1 inhibitors can act either as catalytic inhibitors by DNA intercalation at the Top1 fixation site or as poisons, forming a ternary complex (DNA + Top1 + compound) (24, 25), preventing DNA religation and inducing accumulation of nicked DNA. The addition of proteinase K to the Top1-DNA relaxation test allows the release of nicked DNA that can be resolved and detected on agarose gel. The short half-life of the nicked DNA is stabilized and detectable after addition of a Top1 poison, camptothecin (**Figure 3A**). Nicked DNA was also observed in presence of 0.2 µM of WN197, indicating a Top1 poison activity (**Figure 3A**). At higher concentrations (0.5 µM, 1 µM, 2 µM), the inhibition of Top1 activity without nicked DNA accumulation indicates that WN197 does not act as a Top1 poison.

The effect of WN197 on Top2α and Top2β activities were also assayed. The same principle based on the inhibition of topoisomerase-induced DNA relaxation was used (**Figure 3B**). In the presence of Top2α or Top2β, the supercoiled DNA is relaxed (topoisomers). VP-16 (etoposide, Top2 inhibitor) disturbed DNA relaxation in the reaction, as seen by the presence of supercoiled DNA in the gel, while camptothecin had no inhibitory effect, as expected. WN197 disrupted the Top2α-induced DNA relaxation only at 2 µM, and the Top2β at 1 and 2 µM, higher doses than the concentration necessary to inhibit Top1 activity, indicating a concentration-dependent mechanism of action.

### WN197 Intercalates in DNA

Melting curves and fluorescence measurements were performed to comfort results obtained in **Figure 3**, and ascertain WN197 intercalation in DNA.

Drugs ability to protect calf thymus DNA (CT DNA, 42% GC bp) against thermal denaturation was used as an indicator of the capacity of indenoisoquinoline derivatives to bind and stabilize the DNA double helix. The Cu(II) indenoisoquinoline complex WN197 displayed a slightly higher ΔTm value compared to the metal-free indenoisoquinoline WN170 (respectively 16.6°C and 16.1°C, drug/DNA ratio 0.5), showing a better binding affinity with DNA (**Table 3**).

**Table 3:** Melting curves and fluorescence measurements were determined for WN197 and WN170.

Compound	ΔTm (°C)	Kapp (10^7 M-1)	EtBr displacement
WN197	16.6	15.005 ± 0.290	90%
WN170	16.1	2.436 ± 0.883	87%

Variations in melting temperature (ΔTm=Tm drug−DNA complex−Tm DNA alone) were performed at a ratio of 0.5. Apparent binding constant were measured by fluorescence using [EB]/[DNA] = 1.26. Data were the mean of at least three independent experiments.

The binding affinities, determined using a fluorescence quenching assay based on DNA binding competition between the intercalating drug ethidium bromide and the tested molecules, were used to gain insight into the DNA binding affinity. The apparent DNA binding constant Kapp value of the Cu(II) complex (15.005 ± 0.290 10^7^ M^−1^) is higher compared to the original ligand value (2.436 ± 0.883 10^7^ M^−1^). These results are in agreement with the ΔTm values showing that the complexation of indenoisoquinoline ligand by copper allows a stronger interaction with DNA (**Table 3**).

### WN197 Activates the DNA Damage Response Pathway

**Figure 4:**
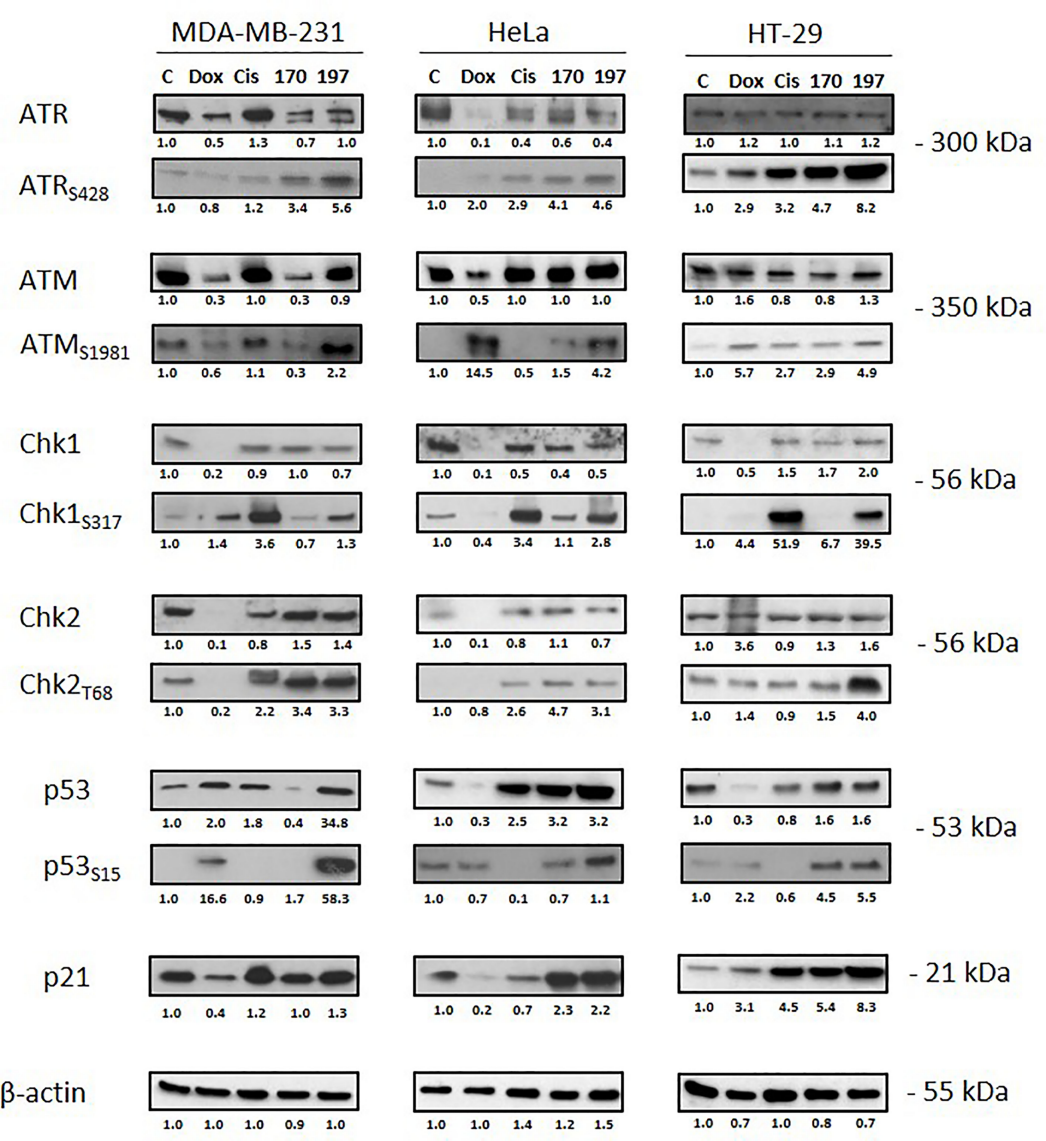
Activation of the DNA Damage Response (DDR) pathway. Cells were treated for 24 h with doxorubicin (5 µM), cisplatin (20 µM), WN170 (0.5 µM), or WN197 (0.5 µM). Western blots were performed to detect ATM, ATR, Chk1, Chk2, p53 and their phosphorylated forms, and p21. β-actin was used as a loading control and relative protein levels were quantified by densitometry using Image J software (Fiji Software, v1.52i). Results were representative of three independent experiments.

The activation of molecular effectors of the DDR pathways involved in SSB and DSB was analysed by Western blot (**Figure 4**). Activating phosphorylation of ATR (S428) and ATM (S1981) occurred in the three cell lines MDA-MB-231, HeLa, and HT-29 treated with WN197 compared to the untreated cells. The subsequent activating phosphorylation of Chk1 (S317) and Chk2 (T68) were observed, confirming the DDR pathway activation. In the doxorubicin, cisplatin, and WN170 these phosphorylations also occurred while in untreated controls they were always lower or absent.

p53 facilitates cell cycle arrest by targeting p21^WAF1/CIP1^. After WN197 treatment, p53 and phosphorylated p53 were increased in MDA-MB-231, HeLa and HT-29 cells (respectively by factors 34.8, 3.2, and 1.6 for p53 and by 58.3, 1.6 and 5.5 for phosphorylated p53), while p21 was highly increased in HT-29 cells (by a factor 8.3) compared to MDA-MB-231 and HeLa (respectively 1.3 and 2.2). The WN170 values are slightly identical except for p53 and p21 in MDA-MB-231 (respectively factors 0.4 and 1.0). In doxorubicin and cisplatin treated cell lines, p53 and p21 were not increased except for p53 in MDA-MB-231 and p21 in HT-29 cells.

### WN197 Induces a Cell Cycle Arrest in G2 Phase

**Figure 5:**
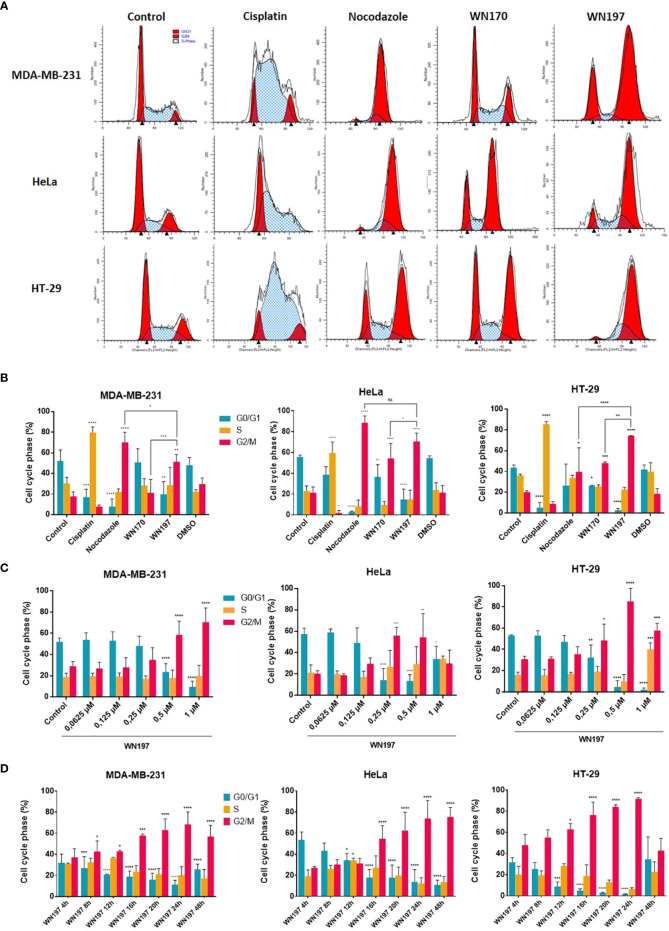
WN197 induced cell cycle accumulation in the G2/M phase. **(A)** Cytograms (G0/G1 and G2/M first and second peaks respectively), and **(B)** flow cytometry analysis of MDA-MB-231, HeLa, and HT-29 cells repartition in the cell cycle 24 h after treatments with cisplatin (20 µM, S phase arrest control), nocodazole (84 nM, M phase arrest control), WN170 or WN197 (0.5 µM). **(C)** Dose-response analysis by flow cytometry of G2/M phase accumulation 24 h after treatments with WN197. **(D)** Time course analysis by flow cytometry of the cell cycle repartition in cell lines untreated (control) or treated with WN197 (0.5 µM). Statistic were based on two-way ANOVA followed by Dunnett’s test (*p<0,05, **p<0,01, ***p<0,005 and ****p<0,001) on three independent experiments.

The cell cycle repartition following the DDR pathway activation was monitored by flow cytometry in cells exposed for 24 h to different treatments (**Figures 5A, B**). Untreated cells showed a classical cell cycle repartition in the 3 cell lines with averages of 50.52% cells in G0/G1 phases, 29.80% in the S phase and 19.68% in the G2/M phases. Cisplatin, known to promote the accumulation of cells in the S phase (57, 58), induced 79.79%, 59.61%, and 85.53% cells in S phase for MDA-MB-231, HeLa, and HT-29 cells, respectively. The mitotic spindle poison, nocodazole, led to an arrest in mitosis with 70.17%, 88.61%, and 39.68% cells in G2/M phase for MDA-MB-231, HeLa, and HT-29, respectively. WN170 did not modified the cell cycle repartition of MDA-MB-231 cells and induced a G2/M accumulation of HeLa and HT-29 cell lines. Treatments with WN197 triggered a G2/M phase accumulation. WN197 had the capacity to induce a higher percentage of cells accumulation in the G2/M phase compared to WN170 respectively with 51.29% and 21.08% for MDA-MB-231 cells, 70.51% and 54.19% for HeLa cells, and 74.4% and 48.06% for HT-29 cells. Sub-G1 peaks were not observed in WN197 treated cells, while they were present after doxorubicin treatment (positive apoptotic control) in **Supplementary Figure S1**.

To determine the lower dose necessary to induce a G2/M phase accumulation, flow cytometry experiments were performed with increasing concentrations of WN197 and results are shown in **Figure 5C**. A G2/M phase accumulation was significantly induced by WN197 from 0.5 to 1 µM for MDA-MB-231, 0.25 to 0.5 µM for HeLa and 0.25 to 1 µM for HT-29.

A kinetic of treatment with WN197 (0.5 µM) was realized on the three adenocarcinoma cell lines by flow cytometry to determine the earliest-induced G2/M accumulation (**Figure 5D**). After 8 h of treatments, the cell cycle was modified for MDA-MB-231 with a significant accumulation in G2/M. A later effect after 12 h and 16 h of treatment was observed respectively for HT-29 and HeLa.

**Figure 6:**
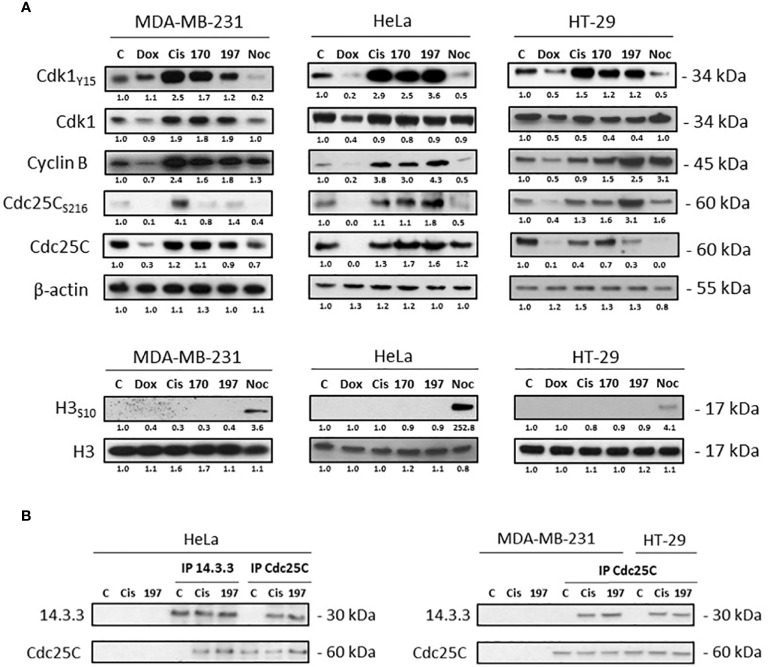
WN197 arrested the cell cycle in G2. **(A)** Western Blot analysis of cells treated for 24 h with doxorubicin (5 µM), cisplatin (20 µM), WN170, WN197 (0.5 µM), or nocodazole (84 nM). β-actin was used as a loading control. For H3 phosphorylation, respective H3 total levels were used as loading controls. **(B)** 14-3-3 and Cdc25C immunoprecipitations were realized in cell lines treated for 24 h with cisplatin (20 µM) or WN197 (0.5 µM). Relative protein levels were expressed by densitometry using Image J software (Fiji Software, v1.52i). Results were representative of three independent experiments.

Cell cycle arrest phase was further determined by Western blot analysis of major cell cycle regulators: Cdk1, cyclin B, Cdc25C phosphatase, and histone H3 (**Figure 6A**). The Cdk1/cyclin B complex that forms the also called MPF (M-phase Promoting Factor) is required for the transition from G2 to M phase of the cell cycle. During the G2/M transition, Cdk1 is activated by dephosphorylation of its threonine 14 and tyrosine 15 residues (inhibitory phosphorylations) by the active Cdc25C phosphatase that requires prerequisite dephosphorylation on threonine 161 (59, 60). In comparison to the untreated control, the phosphorylation of Cdk1 on tyrosine 15 was increased after cisplatin, WN170 or WN197 treatments in the three adenocarcinoma cell lines, while it decreased after treatments with doxorubicin or nocodazole in HeLa and HT-29 and was slightly identical in MDA-MB-231 treated with doxorubicin. The cyclin B amount was increased after WN197 treatment in the three cell lines. Cdc25C was decreased in MDA-MB-231 and HT-29, and increased in HeLa after treatments with WN197 compared to untreated conditions. The inhibitory phosphorylation of Cdc25C on serine 216 was enhanced by WN197 treatments compared to untreated conditions in the three cell lines. On the contrary, a decrease of this phosphorylation was obtained after nocodazole treatments, consistent with the former detection of an activated form of MPF except for HT-29. Finally, histone H3 phosphorylation on serine 10 is involved in mitotic chromatin condensation and is a marker for entry in the M phase after activation of the Cdk1/Cyclin B complex (61). In WN197 treated cells, histone H3 was not phosphorylated on serine 10, showing that cancer cells were stopped in the G2 phase before they could reach the M phase. On the contrary in nocodazole treated adenocarcinoma lines in which an arrest in the M phase occurs, histone H3 was phosphorylated on serine 10.

Furthermore, as seen in **Figure 6B**, Cdc25C phosphorylated on serine 216 was trapped by 14-3-3 as shown by Cdc25C or 14-3-3 immunoprecipitations realized in HeLa, and Cdc25C immunoprecipitations in MDA-MB-231 and HT-29 cells after 24 h of treatment with 0.5 µM of WN197. The binding was observed after cisplatin treatment but not in untreated controls.

### WN197 Induces Autophagy

**Figure 7:**
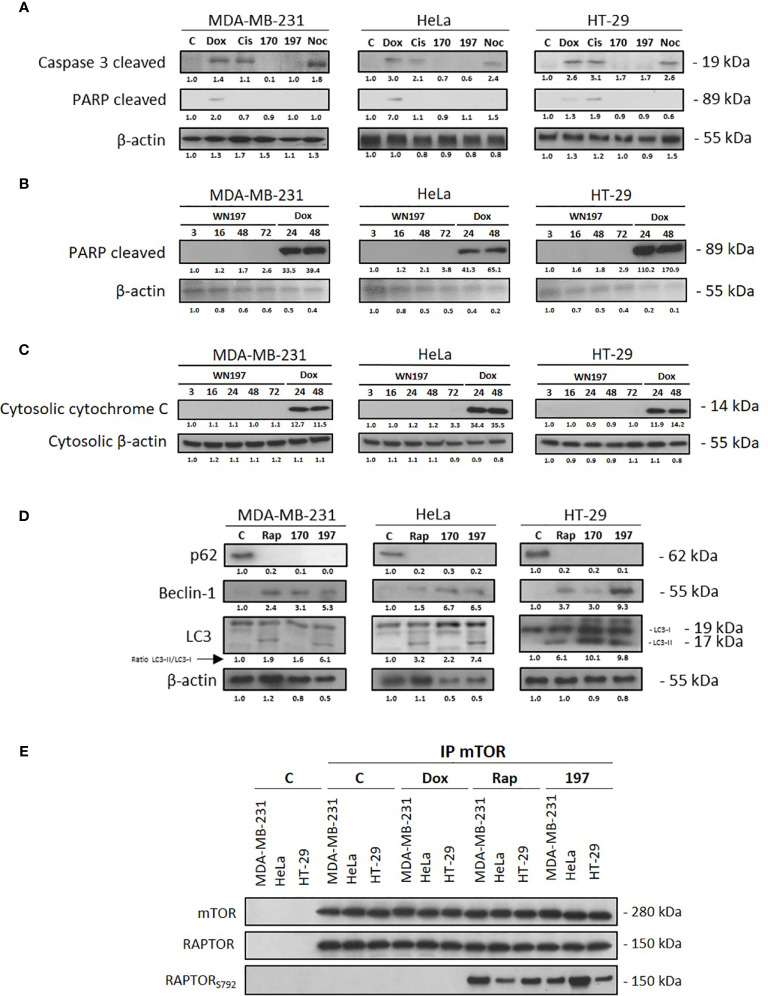
WN197-induced autophagy. Cells were treated for 24 h with doxorubicin (5 µM), cisplatin (20 µM), WN170, WN197 (0.5 µM), nocodazole (84 nM) or rapamycin (0.5 µM). **(A)** Cleaved caspase 3 and PARP analysis by Western blots. Western blot analysis, after 3, 16, (24 or not), 48 and 72 h of treatment with WN197 or doxorubicin for 24 and 48 h, of **(B)** cleaved PARP or **(C)** cytosolic cytochrome **(C, D)** p62, Beclin-1, and LC3 markers analysis by Western blot. LC3 levels were expressed upon the LC3-II/LC3-I ratio. β-actin levels were used as a loading control. Relative protein levels were expressed by densitometry using Image J software (Fiji Software, v1.52i). **(E)** mTOR immunoprecipitations were realized in cell lines untreated or treated with doxorubicin (5 µM), rapamycin (0.5 µM) or WN197 (0.5 µM) for 24 h and followed by Western blots.

Apoptosis is often activated after DNA damage (25, 62). However, the early apoptosis marker cleaved caspase 3 and the late apoptosis marker cleaved PARP were not detected after treatments with WN197 and WN170 in contrast to doxorubicin and cisplatin treatments (**Figure 7A**). A time-course detection of cleaved PARP and cytochrome C release in the cytoplasm at 3, 16, 24, 48, and 72 h compared to doxorubicin apoptosis positive control at 24 and 48 h (**Figures 7B, C**) and annexin V tests (**Figure S2**) confirm apoptosis is not triggered by WN197. These data indicate that apoptosis is not the programmed cell death activated.

We then determined whether WN197 and WN170 could induce autophagy. In the three adenocarcinoma cell lines, several autophagy markers (63) were detected. p62/sequestosome-1 was degraded, Beclin-1 was synthesized and LC3-I association with phosphatidyl-ethanolamine that forms LC3-II was increased as shown by accumulation of LC3-II after 24h of treatment with 0.5 µM of WN197 and WN170 (**Figure 7D**). The same changes were observed with the inhibitor of mTOR pathway, rapamycin which is known to activate the autophagy process. Moreover, immunoprecipitation carried on the mTOR complex showed that the RAPTOR component was phosphorylated on serine 792 after treatment with 0.5 µM of WN197, as seen in positive controls treated with 500 nM of rapamycin, and compared to negative controls treated with doxorubicin (**Figure 7E**).

## Discussion

This study aims to develop and understand the molecular properties of a new organometallic compound WN197, derived from the topoisomerase 1 inhibitor indenoisoquinoline. Previous studies highlighted action specifically correlated to the presence of a metallic atom like copper (53), iron [e.g. ferrocen/ferroquine (43, 64)], ruthenium [e.g. indenoisoquinoline (55) and various complexes (65, 66)], or platin [e.g. cisplatin (67)], and demonstrate the interest of these organometallic compounds in cancerology. More recently, a class of topoisomerase inhibitor, the indenoisoquinoline derivatives, were developed and selected for their high stability and non-drug substrate for efflux transporters involved in cell resistance (35, 68). These promising compounds are in phase I/II clinical trials (36, 68). However, constant efforts are made to increase their efficiency. The addition of a carbohydrate moiety to indenoisoquinoline derivatives significantly improves the binding affinity to DNA due to a stronger interaction through hydrogen bonds (69). Hereby, we synthesised a new copper indenoisoquinoline derivative. The copper(II) addition to the indenoisoquinoline backbone significantly enhance the toxicity on triple-negative breast MDA-MB-231 and cervix HeLa cancer cell lines. Those two cell lines are related to breast and cervix cancers with high mortality rates in women. In addition, the toxicity is obtained at lower doses compared to human non-tumorigenic epithelial cell line MCF-10A. The use of low doses in chemotherapy could be of particular interest and represent an advantage with less risk of adverse side effects. Further experiments will help to determine if WN197 has specificity at the cellular level.

The viability assays showed that low doses are necessary to induce cell death in breast, cervix, and colon cancer cell lines, from three of the most prevalent adenocarcinomas. The IC_50_ are under the values obtained for most other Top1 inhibitors that usually range from concentration of 1 to 10 μM except for thiosemicarbazone or pyrimidine-derived compounds (53). The medium value of 0.5 μM, close to the IC_50_ for the three adenocarcinoma cell lines, was further chosen to decipher the molecular pathways involved in the anti-proliferative effect of WN197. Topoisomerases are overexpressed in M phase in cancer cells and generate a high number of DNA breaks under the action of Top inhibitors (12, 14, 15). Cells overexpressing topoisomerases have shown better responses to Top inhibitors (70, 71). Using low doses of the compound could be useful to avoid unwanted normal cell death. Such strategies of low minimal but necessary anti-tumorigenic doses are often employed for anthracycline to limit cardiotoxicity (72, 73).

We determined the extent of DNA damage induced by the new compound, with immunofluorescence and Western blot analysis of a front-line activated marker of DNA breaks, the γH2AX histone. The recruitment of γH2AX normally occurs at the site of DNA breaks after exposition to Top1 or Top2 poisons (74, 75). Higher level of DNA breaks is observed with WN197 compared to the control copper-free compound WN170, proving that the presence of a metal atom increases the efficiency to induce DNA damage. DNA breaks appear early around 30 min after addition of the product. In parallel, *in vitro* tests reveal that WN197 inhibits Top1 at low doses, corresponding to the IC_50_, and Top2 at higher doses up to 1 μM showing a dose-dependent action. The copper complex WN197 is a Top1 poison that forms a ternary complex with the DNA (interfacial inhibition) as indenoisoquinoline derivatives (24).

After DNA damage is induced, DDR effectors are activated, as shown in Western blot experiments. The upstream kinases ATM, ATR, Chk1, and Chk2 are phosphorylated after 24 h of treatment with 0.5 µM of WN197, a prerequisite for their activation (76, 77). Both SSB (ATR, Chk1) and DSB (ATM, Chk2) markers are detected at a concentration capable to inhibit Top1. Top1 are known to generate SSB and Top2 DSB. However, Top1 poisons produce SSB that can be converted into DSB, the most dangerous type of DNA break, at the replication fork stalling (78, 79) explaining the activation of both SSB and DSB markers in our experiments. The cell cycle arrest induced by 0.5 µM of WN197 occurs in the G2/M phase for all cancer cell lines analysed, as early as 8 h or 16 h with a maximal number of arrested cells after 24 h of treatment and is maintained at 48 h. Concentration values ranging from 0.25 µM to 1 µM of WN197 are necessary to trigger the G2/M arrest. This result is consistent with the dose-dependent inhibitory effect obtained in the *in vitro* topoisomerase inhibition tests where Top1 inhibition is obtained with values between 0.2 µM and 0.5 µM. Above 1 µM a different DNA migration profile is detected showing WN197 poison activity is lost for a different type of inhibition. A catalytic mode of inhibition could occur through intercalation of WN197 into DNA. At doses above 1 µM, the compound exerts a dual Top1/Top2 inhibitory activity and intercalation properties as demonstrated by the melting curves and the fluorescence measurements. The planar indenoisoquinoline skeleton of WN197 displays an increased intercalation into DNA compared to WN170. The high affinity of the Cu(II) complex with DNA can be attributed to the π-cation interaction between the base pairs and the atom of Cu(II) coordinated with ligands, but also to the capability to increase the π-π interaction between the base pairs of DNA and a second ligand molecule (80, 81). At high doses, DNA intercalation could avoid topoisomerase access to its fixation site similarly to a catalytic inhibitor. Such mechanism is found with anthracyclines such as doxorubicin whose poison activity at low doses is lost for an intercalating catalytic inhibitory activity at high doses. Due to a strong affinity for DNA duplexes, those anthracycline compounds prevent Top2 binding to DNA (75, 82).

To determine the exact arrest phase in the cell cycle, analyses were further conducted. To allow the G2 to M phase transition, Cdc25C dephosphorylates on residues tyrosine 15 and threonine 14, leading to its activation (83, 84). Cdk1 activation in the MPF complex phosphorylates histone H3 on serine 10 to allow DNA condensation during mitosis (61). After 24 h of treatments, an increase in the inhibitory serine 216 phosphorylation of Cdc25C is detected. This phosphorylation is recognized by 14-3-3 (85) to form a complex with Cdc25C, as shown in the three adenocarcinomas, by immunoprecipitation. Sequestration of Cdc25C by 14.3.3 impedes Cdk1 dephosphorylation on tyrosine 15 and histone H3 phosphorylation does not occur on serine 10 in the three cell lines after treatment with WN197 for 24 h. The cancer cell lines lack the required MPF activation and H3 phosphorylation to allow an M phase entry and remain arrested in G2. In addition, cyclin B accumulates in our experiments concomitantly and is not destroyed by the proteasome as expected at the end of the M phase (86, 87). p53 and its target the cell cycle inhibitor p21 are increased after WN197 treatments. p53 is involved in cell-cycle arrest by a transcriptional activation of p21 capable to inhibit Cdk1/cyclin B and cell-cycle progression through mitosis (88–90). p53 also targets 14-3-3 and blocks G2/M transition (91). Altogether, the results demonstrate that WN197 at low doses with a Top1 poison activity arrest adenocarcinoma cells in G2. After DNA damage have been induced, activation of the DDR pathways normally ensures repairs but when damage is too extended, cells undergo a programmed death (92, 93). While most of the actual topoisomerase inhibitors induce apoptosis (25, 62), WN197 triggers autophagy. Among topoisomerase I inhibitors, a camptothecin derivative irinotecan and an indenoisoquinoline compound NSC706744 were reported to activate autophagy with the absence of apoptosis (94, 95). After 24 h of treatment with low doses of WN197 (0.5 μM), autophagy markers are detected by Western blots: synthesis of Beclin-1 (96), increase in LC3-II/LC3-I ratio (97), and degradation of p62 (98). It was previously shown, after DNA damage, that the mTORC1 complex was inhibited by RAPTOR phosphorylation (on multiple sites including serine 792) in a negative feedback loop to induce autophagy (99, 100). We further show autophagy is triggered through the phosphorylation of RAPTOR in the mTOR complex. This mechanism of activation is similar to the mTORC1 inhibitor rapamycin (101). Our results show that under WN197 treatment from 3 to 72 h, cells die by a caspase-independent mechanism as classical markers annexin V staining, caspase 3 and PARP cleavage, cytoplasmic cytochrome C released were not detected. It also has to be noted, no sub-G1 cells were detected after WN197 treatment while they were after doxorubicin known to induce apoptosis. Previous data on breast cancer cells have showed autophagy could mask and delay apoptosis but was associated with an early release of cytochrome C from mitochondria which is not the case in our experiments (102). Cytochrome C is not released when autophagy is triggered and mitochondria degraded in autophagosomes (103). Several studies have described autophagy as dependent on wild-type p53 depletion or inhibition (104). WN197 action is associated with an increase in p53 and p53 phosphorylation. However, the induced-autophagy does not dependent on the cell lines p53 status. HeLa cells express wild-type p53 that end up as functionally null when targeted to degradation by E6 endogenous papillomavirus protein, while MDA-MB-231 and HT-29 display p53 mutations resulting in positive gain of function (105). Nevertheless, WN197 induced-autophagy is in agreement with an increase of p21 level and the G2 arrest detected our experiments in cancer cells. Several anti-apoptotic effects of p21 can explain the choice of an autophagic cell death instead of apoptosis. High levels of p21 are known to block Cdk1/cyclin B and to inhibit apoptosis through down-regulation of caspase-2 (106), stabilization of anti-apoptotic cellular inhibitor of apoptosis protein-1, c-IAP1 (107), and inhibition of procaspase 3 activity (108). Another additional mechanism through Beclin-1 could play an important role in apoptosis inhibition and autophagy. Beclin-1 protein expression was shown necessary to block the apoptotic cascade after induced-DNA damage (102, 109) and to activate autophagy under low doses of chemotherapeutics (rapamycin, tamoxifen) in breast and ovarian cancers (110, 111).

## Conclusion

**Figure 8:**
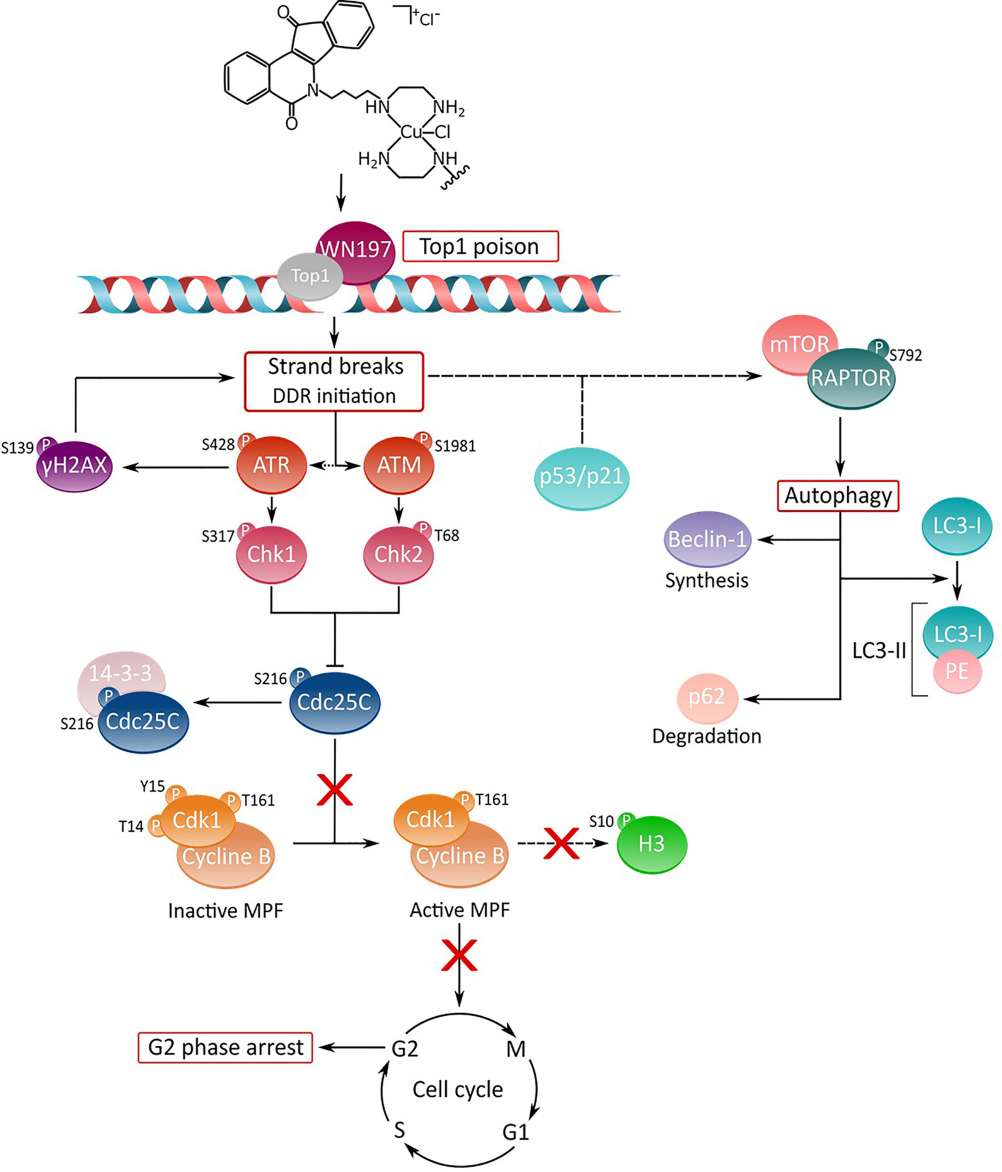
Deciphering of the molecular mechanisms of the novel copper(II) indenoisoquinoline complex WN197. WN197 inhibits topoisomerases I at low doses in a poison mode and forms a ternary complex with the topoisomerase and DNA, leading to strand breaks accumulation. Phosphorylated H2AX (γH2AX) localizes at the sites of DNA damage. The DNA damage response pathway is activated: ATM and ATR kinases are phosphorylated, and subsequently activate Chk1 and Chk2, leading to Cdc25C phosphorylation on serine 216 (S216), and to its binding to 14-3-3. Consequently, Cdk1 remains phosphorylated on tyrosine 15 (Y15), impeding the activation of the MPF (Cdk1/Cyclin B) and the phosphorylation of H3 on serine 10 (S10). Cancer cells arrest in the G2 phase of the cell cycle. The DDR also leads to an increase in p53 and p21 followed by an autophagic cell death characterized by the phosphorylation of RAPTOR on serine 792 (S792) in the mTORC1 complex, the synthesis of Beclin-1, the formation of LC3-II (complex LC3-I/PE (phosphatidylethanolamine)), and the degradation of p62.

Copper(II) indenoisoquinoline complex WN197 displays an anti-cancerous activity at low doses inhibiting Top1. MDA-MB-231 (triple negative breast cancer cells), HeLa (cervix cancer cells), and HT-29 (colon cancer cells), cancer cells accumulate DNA breaks and arrest in the G2 phase of the cell cycle. This arrest is characterized by the inactivation of the Cdc25C phosphatase through phosphorylation on serine 216 and binding to 14.3.3 that consequently leaves in its inactive form the MPF (a phosphorylated form of Cdk1 associated to accumulated cyclin B). Autophagy is further processed by the RAPTOR effector phosphorylation in the mTOR complex, and associated to p21 overexpression. WN197 appears as a new efficient drug to counteract cancer cells when used at low doses. The action mechanism of the copper complex is summarized in **Figure 8**. Its use in chemotherapy could particularly benefit patients with cancer cells overexpressing topoisomerases or sensitize cancer cells to other DNA modifying agents including DNA adducts inducer, methylating agents, or PARP inhibitors (112, 113).

## Data Availability Statement

The raw data supporting the conclusions of this article will be made available by the authors, without undue reservation.

## Author Contributions

Conceptualization: CM, LP, KC, and AM. Performing experiments: CM, NW, TB, LP, KC, and AM. Manuscript reviewing: A-SV-E. Writing and editing: CM, LP, KC, and AM. All authors contributed to the article and approved the submitted version.

## Funding

CM is a recipient of a doctoral fellowship from the French ministry. This work was supported by the CNRS, the University of Lille, and by grants from the “Ligue Contre le Cancer, Comités Nord et Aisne” (AM).

## Conflict of Interest

Author NW was employed by AGAT Laboratories, Intertek.

The remaining authors declare that the research was conducted in the absence of any commercial or financial relationships that could be construed as a potential conflict of interest.

## Publisher’s Note

All claims expressed in this article are solely those of the authors and do not necessarily represent those of their affiliated organizations, or those of the publisher, the editors and the reviewers. Any product that may be evaluated in this article, or claim that may be made by its manufacturer, is not guaranteed or endorsed by the publisher.

## Supplementary Material

Supplementary Figure 1 Cytograms obtained after flow cytometry analysis of MDA-MB-231 cells 24 h after treatments or not with WN197 (0.5 µM) or apoptosis positive control doxorubicin (5 µM, showing sub-G1 accumulation).

Supplementary Figure 2 Detection of apoptosis feature by annexin V-propidium iodide (PI). MDA-MB-231, HeLa, and HT-29 cells were cultivated to 80% of confluence, incubated or not for 24 h with WN170 (0.5 µM), WN197 (0.5 µM), camptothecin (20 µM; CPT) or doxorubicin (5 µM; Doxo), trypsinized, and washed in ice-cold PBS. Cell suspensions were treated with PI and annexin V-FITC reagent (Apoptosis Detection Kit, BD) using the manufacturer’s protocol before they were analysed by flow cytometry **(**CytoFLEX LX, Beckman Coulter**)** with Kaluza analysis software (v2.1.1). **(A)** Y-axis: number of PI-stained cells. X-axis: number of annexin V-FITC-stained cells. The lower left quadrant represents non-apoptotic cells (annexin V-FITC-negative and PI-negative cells; B–), the lower right quadrant represents early apoptotic cells (annexin V-FITC-positive and PI-negative cells; B+-), the upper right quadrant represents late apoptotic/necrotic cells (annexin V-FITC-positive and PI-positive cells; B++), and the upper left quadrant represents prenecrotic cells (annexin V-FITC-negative and PI-positive cells; B-+). **(B)** Representative histograms. Camptothecin and doxorubicin induced apoptosis in the three cancer cell lines, while WN170 and WN197 had no effect compared to the control.

## Abbreviations

ATM, ataxia telangiectasia mutated; ATR, ataxia telangiectasia related; BSA, bovine serum albumin; Cdc25, cell division cycle 25; Cdk1, cyclin dependent kinase 1; Chk1/2, checkpoint kinases 1/2; c-IAP1, cellular inhibitor of apoptosis protein 1; DDR, DNA damage response; DNA-PK, DNA-dependent protein kinase; DSB, double strand break; H2AX, H2A histone family member X; IC_50_, half maximal inhibitory concentration; Kapp, apparent dissociation constant; mTOR, mammalian target of rapamycin; MTS, 3-(4,5-dimethylthiazol-2-yl)-5-(3-carboxymethoxyphenyl)-2-(4-sulfophenyl)-2H-tetrazolium; _N_DNA, nicked DNA; PBS, phosphate-buffered saline; RAPTOR, regulatory-associated protein of mTOR; _R_DNA, relaxed DNA; _SC_DNA, supercoiled DNA; SDS, sodium dodecyl sulfate; SSB, single strand break; t-AML, therapy-related acute myeloid leukemia; TLC, thin-layer chromatography; Tm, temperature of melting; Top, Topoisomerase; VP-16, etoposide.
