# Supplementary figures and images for: A Novel Copper(II) Indenoisoquinoline Complex Inhibits Topoisomerase I, Induces G2 Phase Arrest, and Autophagy in Three Adenocarcinomas
Source: Front Oncol. 2022 Feb 24;12:837373. doi: 10.3389/fonc.2022.837373 (PMC8908320; doi:10.3389/fonc.2022.837373)

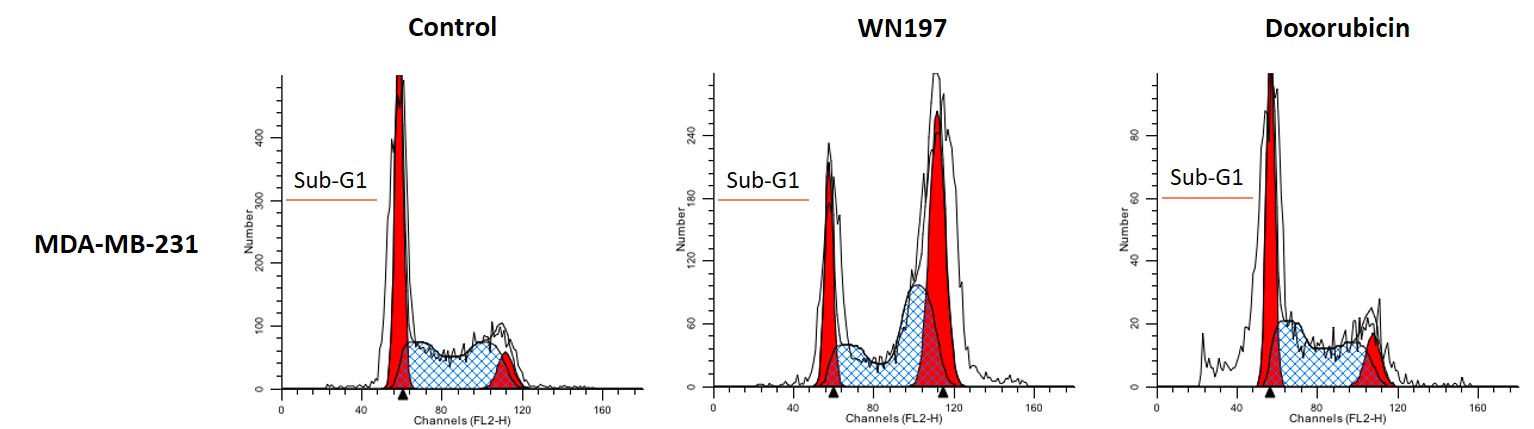

Supplement: Supplementary Figure 1 — Cytograms obtained after flow cytometry analysis of MDA-MB-231 cells 24 h after treatments or not with WN197 (0.5 µM) or apoptosis positive control doxorubicin (5 µM, showing sub-G1 accumulation). [file Image_1.jpeg]

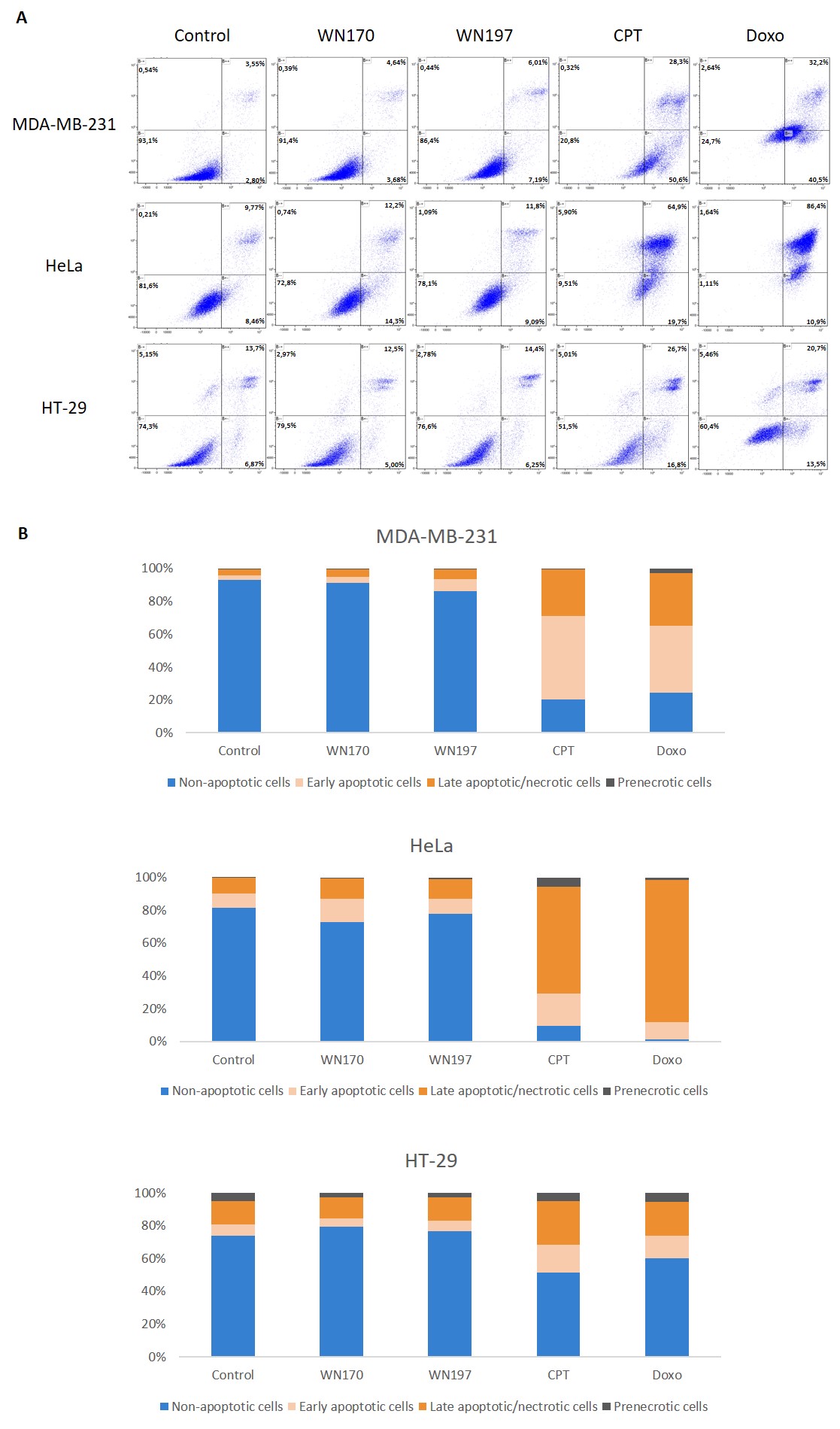

Supplement: Supplementary Figure 2 — Detection of apoptosis feature by annexin V-propidium iodide (PI). MDA-MB-231, HeLa, and HT-29 cells were cultivated to 80% of confluence, incubated or not for 24 h with WN170 (0.5 µM), WN197 (0.5 µM), camptothecin (20 µM; CPT) or doxorubicin (5 µM; Doxo), trypsinized, and washed in ice-cold PBS. Cell suspensions were treated with PI and annexin V-FITC reagent (Apoptosis Detection Kit, BD) using the manufacturer’s protocol before they were analysed by flow cytometry (CytoFLEX LX, Beckman Coulter) with Kaluza analysis software (v2.1.1). (A) Y-axis: number of PI-stained cells. X-axis: number of annexin V-FITC-stained cells. The lower left quadrant represents non-apoptotic cells (annexin V-FITC-negative and PI-negative cells; B–), the lower right quadrant represents early apoptotic cells (annexin V-FITC-positive and PI-negative cells; B+-), the upper right quadrant represents late apoptotic/necrotic cells (annexin V-FITC-positive and PI-positive cells; B++), and the upper left quadrant represents prenecrotic cells (annexin V-FITC-negative and PI-positive cells; B-+). (B) Representative histograms. Camptothecin and doxorubicin induced apoptosis in the three cancer cell lines, while WN170 and WN197 had no effect compared to the control. [file Image_2.jpeg]
